# N-polar GaN Film Epitaxy on Sapphire Substrate without Intentional Nitridation

**DOI:** 10.3390/ma15093005

**Published:** 2022-04-21

**Authors:** Zhaole Su, Yangfeng Li, Xiaotao Hu, Yimeng Song, Rui Kong, Zhen Deng, Ziguang Ma, Chunhua Du, Wenxin Wang, Haiqiang Jia, Hong Chen, Yang Jiang

**Affiliations:** 1 Key Laboratory for Renewable Energy, Beijing Key Laboratory for New Energy Materials and Devices, Beijing National Laboratory for Condensed Matter Physics, Institute of Physics, Chinese Academy of Sciences, Beijing 100190, China; zhaole_su@iphy.ac.cn (Z.S.); liyangfeng12@mails.ucas.ac.cn (Y.L.); huxiaotao16@ucas.ac.cn (X.H.); zhen.deng@iphy.ac.cn (Z.D.); zgma@iphy.ac.cn (Z.M.); duchunhua@iphy.ac.cn (C.D.); wxwang@iphy.ac.cn (W.W.); mbe2@iphy.ac.cn (H.J.); hchen@iphy.ac.cn (H.C.); 2 Center of Materials and Optoelectronics Engineering, University of Chinese Academy of Sciences, Beijing 100049, China; 3 Beijing Key Laboratory for Magneto-Photoelectrical Composite and Interface Science, School of Mathematics and Physics, University of Science and Technology Beijing, Beijing 100083, China; b20180340@ustb.edu.cn; 4 School of Microelectronics, Nantong University, Nantong 226019, China; hgao@iphy.ac.cn; 5 The Yangtze River Delta Physics Research Center, Liyang 213000, China; 6 Songshan Lake Materials Laboratory, Dongguan 523808, China

**Keywords:** N-polar GaN, MOCVD, nitriding, AlN

## Abstract

High-temperature nitridation is commonly thought of as a necessary process to obtain N-polar GaN films on a sapphire substrate. In this work, high-quality N-polar GaN films were grown on a vicinal sapphire substrate with a 100 nm high-temperature (HT) AlN buffer layer (high V/III ratio) and without an intentional nitriding process. The smallest X-ray full width at half maximum (FWHM) values of the (002)/(102) plane were 237/337 arcsec. On the contrary, N-polar GaN film with an intentional nitriding process had a lower crystal quality. In addition, we investigated the effect of different substrate treatments 1 min before the high-temperature AlN layer’s growth on the quality of the N-polar GaN films grown on different vicinal sapphire substrates.

## 1. Introduction

Nitrogen-polar (N-polar) GaN is attracting more attention because of its multiple potentials in reducing efficiency droop and the threshold current density of light-emitting diodes (LEDs) [1], enhancing the incorporation of indium into InGaN multiple quantum wells (MQWs) [2], etc. N-polar GaN film has successfully been grown on various foreign substrates such as sapphire, Si, and SiC [3,4,5]. For N-polar GaN film grown on sapphire substrate, high-temperature (>700°) nitridation has been considered as a necessary step to obtain N polarity, and this nitriding process has a critical effect on the quality of subsequent N-polar GaN film [6]. Several groups found that N-polar GaN covered with hexagonal islands could be obtained when a high-temperature (>1000 °C) AlN buffer layer was applied directly on a C-plane sapphire substrate without an intentional nitriding process [7,8]. They attributed this polarity conversion to unintentional nitriding at the initial stage of high-temperature AlN layer growth. However, they stopped here without further optimization to obtain better N-polar GaN films. Other groups also found that excessive nitriding (extra high-temperature or excessive nitriding time) could deteriorate crystal quality of N-polar GaN film [9]. In the precondition of intentional nitriding, a nitriding temperature of 900~1000 °C is considered to be the optimal window for obtaining high-quality N-polar GaN films [6,7,8,9,10]. In this work, we applied a temperature as high as the AlN buffer layer (1050 °C) to nitride the sapphire substrate for 1 min. Innovatively, different flow rates of the TMAl source were also inlet into the reaction chamber simultaneously during the nitriding process. Ultimately, a better quality N-polar GaN film was obtained under the condition of nitriding with NH_3_ and a certain amount of TMAl simultaneously, other than that with an intentional nitriding process. This phenomenon provides deeper insight into the effect of the high-temperature nitriding process on the polarity of GaN film grown on a sapphire substrate.

## 2. Experiment

The N-polar GaN films were grown by the Aixtron close-coupled showerhead (CCS) MOCVD. Trymethylgallium (TMGa) and trymethylaluminum (TMAl) were used as group III sources, ammonia (NH_3_) was used as a group V source, and hydrogen was used as a carrier gas. In every growth experiment, there were three different types of sapphire substrates simultaneously (i.e., on-axis C plane, 2° off-cut towards the M-axis, and 4° off-cut towards the A-axis, labeled as C, 2M, and 4A, respectively). The substrates were first baked for 7 min at a temperature of 1060°; during the next 1 min, three methods were applied to treat the sapphire substrates to obtain the initial N polarity at a temperature of 1050 °C. Specific NH_3_ and TMAl source flow of each treatment method and corresponding sample labels are shown in Table 1. It could easily be seen that the A samples underwent a commonly intentional nitriding process, but the B and C samples were treated with both NH_3_ and a TMAl source (i.e., unintentional nitridation). This 1 min process (intentional or unintentional nitridation) is collectively called the nitriding process. Then, a 100 nm AlN buffer layer was grown at a pressure of 100 mbar and a temperature of 1050 °C. The V/III ratio of the AlN buffer layer was the same as that of the 1 min nitriding process of the B samples. Namely, the nitriding process for the B samples was indistinguishable from the growth of the HT AlN buffer layer; therefore, it can also be regarded as growing directly on the sapphire substrate without intentional nitridation. Finally, a GaN layer with a total thickness of 1.5 µm was grown with two different growth rates at the temperature of 1020 °C.

The growth process of these samples was monitored by real-time in situ reflectance; the surface morphology of the samples was characterized by optical microscopy (OM), scanning electron microscopy (SEM), and atomic force microscopy (AFM); the crystal quality was characterized by HRXRD and room-temperature photoluminescence spectroscopy (PL).

## 3. Results and Discussion

**Figure 1:**
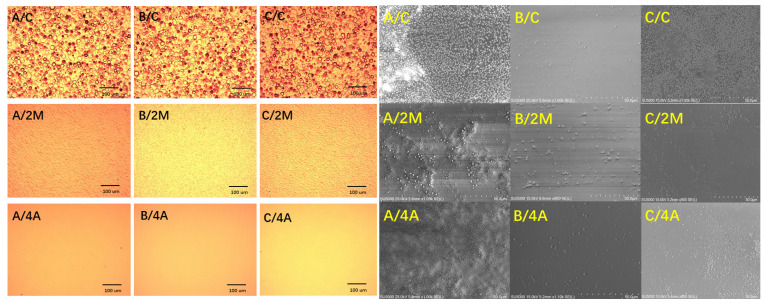
Optical microscope images of all samples before KOH etching and SEM images of all samples after KOH etching in which the form of the sample/substrate is marked.

The OM images of samples A–C with different vicinal substrates are shown in Figure 1. Micron-level hexagonal pyramids appeared on the surface of the GaN films grown on C substrate, which is the typical morphology of N-polar GaN films. For GaN films grown on 2M substrate, the hexagonal pyramids disappeared, and many wavy swellings were seen on the surface. The surface of GaN films grown on 4A substrate were mirror-like smooth. This can be explained by the fact that the Ga adatom on the N-polar surface had a lower diffusion than the Ga-polar, and the steps formed by beveling the sapphire substrate promoted a step-flow growth mode to suppress hexagonal islands [4].

In this study, the polarity was examined by hot (80 °C) KOH solution etching during which N-polar GaN was reactive but Ga-polar was resistant to alkaline solution [10]. The SEM images of samples A–C, grown on different substrates after KOH etching, are shown in Figure 1. It can be seen that all of the samples were almost completely etched, and only some hexagonal islands of Ga polarity remained on the surface. This is the typical surface morphology of N-polar GaN films after KOH etching [11].

**Figure 2:**
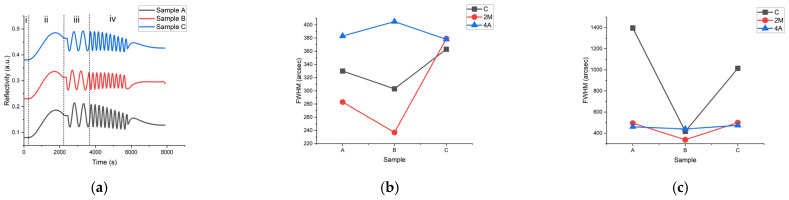
The in-situ reflectivity of 2M of three samples: A, B, and C. Stage (i): baking and initial treatment of the substrate; stage (ii): the high-temperature AlN layer; stage (iii): low-speed GaN; stage (iv): high-speed GaN. (**a**) XRD FWHM results for samples A, B, and C at approximately a (002) reflection (**b**) and a (102) reflection, and (**c**) grown on C, 2M, and 4A sapphire substrates.

In every experiment, only the N-polar GaN layer grown on 2M substrate were monitored by in situ reflectivity (the laser wavelength was 633 nm) as shown in Figure 2a. There were four stages during the total growth. At stage (i), the substrates were baked and then were conducted with intentional or unintentional nitriding for 1 min; the reflectivity remained unchanged. At stage (ii), a 100 nm high-temperature AlN buffer layer was grown, and the reflectivity oscillated by an approximately two-third period. At stage (iii), a 300 nm GaN layer was grown with a low growth rate of 0.92 µm/h. It was observed that the amplitude of the second period was larger than for the first of sample C. However, for samples A and B, this phenomenon was opposite, and the second amplitude was smaller. This may have been caused by different levels of nitriding so that the surface of the AlN layer of sample C was rougher than that of samples A and B. At stage (iv), the growth rate increased to 2.14 µm/h, the amplitude began to diminish, and the center of oscillation began to decline owing to the roughening of the surface. There was a difference in that the declining level of sample B was minimal compared with samples A and C, which implied the best crystal quality and surface roughness.

To obtain the crystal quality of samples A–C with different vicinal substrates, the X-ray (002) and (102) diffraction rocking curves and the FWHM results were obtained as shown in Figure 2b,c. For samples grown on C and 2M sapphire substrates, sample B had the smallest FWHM values for both the (002) and (102) rocking curve, but the (002) FWHM of sample B became larger than for samples A and C grown on 4A sapphire substrate. Comparing the samples with different substrates, the highest quality films in samples B and C were those grown on 2M and 4A substrate, respectively. For sample A, the film with the smallest (002) FWHM value was that grown on 2M substrate, and the film grown on 4A substrate had the smallest (102) FWHM value. Sample B grown on a 2M sapphire substrate without intentional nitriding process had the smallest (002) and (102) FWHM values of 237 arcsec and 337 arcsec, respectively.

**Figure 3:**
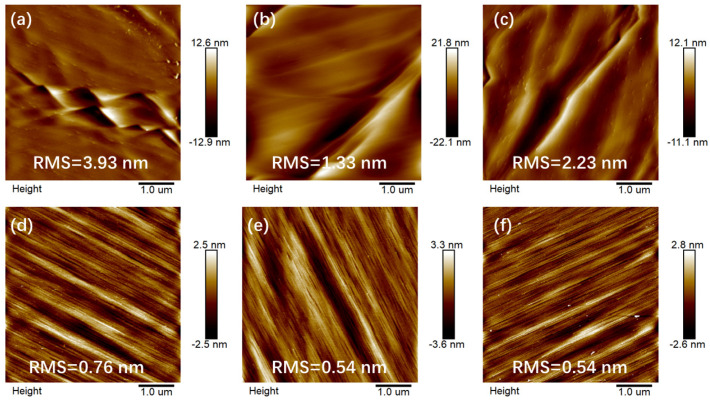
The 5 × 5 µm^2^ AFM images of samples A (**a**,**d**); B (**b**,**e**); C (**c**,**f**) grown on 2M (**a**–**c**) and 4A (**d**–**f**) substrates.

The surface roughness values of the different samples were obtained by AFM as shown in Figure 3. As the surface of the N-polar GaN films grown on C substrate was covered with hexagonal pyramids, the AFM measurement was meaningless (surface undulation reaches micro level). Thus, only the surfaces of samples grown on 2M and 4A substrates were measured. For N-polar GaN films grown on 2M substrate, the surface had a few jagged bumps on account of low diffusion. However, the surface of N-polar GaN films grown on 4A substrate showed many steps, and the RMS roughness decreased significantly. It was seen that the surface RMS roughness of sample B grown on 2M substrate was the smallest. From the RMS roughness results, it can be observed that the surface of sample B was the smoothest.

**Figure 4:**
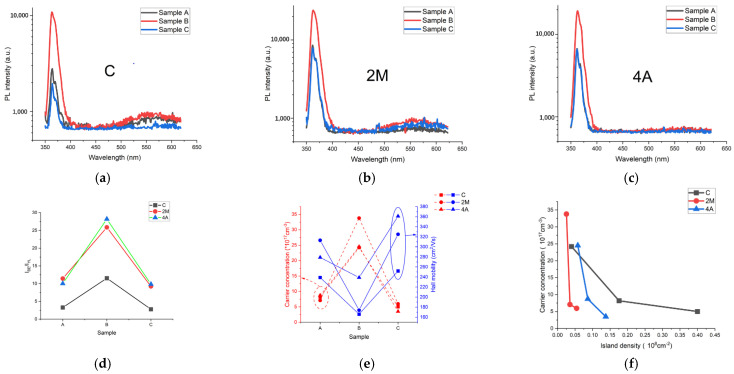
Room-temperature PL results of samples A, B, and C grown on C: (**a**); 2M: (**b**); 4A: (**c**); substrate. I_BE_/I_YL_ results of all samples: (**d**). Summary of the Hall measurement results: (**e**) and the relation between carrier concentration and island density formed after KOH etching: (**f**).

To obtain the details of their optical property, the total samples were measured by room-temperature PL (laser wavelength was 325 nm), and the results are shown in Figure 4. The near band emission (BE) peaks were conspicuous at a wavelength of 366 nm for all samples. The yellow luminescence (YL) bands were also distinct with a center wavelength of 560 nm. The mechanism of the YL is still under discussion. Currently, it is generally accepted that YL is caused by the transition of electrons from the conduction band or shallow donor level to the deep acceptor level [12,13,14,15]. The shallow donor is mostly an O_N_ defect, while the deep acceptor is provided by V_Ga_ (unintentional doping) or C_N_ (high resistance by C doping) defects [16,17,18,19,20]. It can be seen that the YL intensity had a great relationship with the substrate, and the intensity of YL band follows a decreasing trend: C > 2M > 4A. For different samples, the sample B not only had the strongest BE intensity but also the strongest YL intensity, which was related to the highest unintentional doping carrier concentration because there were more possibilities for electron transition. The ratios of BE intensity to YL intensity (I_BE_/I_YL_) are summarized in Figure 4d. The I_BE_/I_YL_ value can be seen as a variable to characterize the deep acceptor concentration level, which strips out the effect of carrier concentration, and the deep acceptor (V_Ga_) concentration for sample B was the lowest. From the results, we can conclude that B samples had the best optical performance.

To obtain the electrical property of the N-polar GaN films, Hall measurements were executed, and the results are shown in Table 2 and Figure 4e. It is interesting that the unintentional doping carrier concentrations for the B samples were higher than for samples A and C and that sample C had the lowest for every substrate. Although the carrier concentration of the B samples was the highest, the value was in the same order of magnitude as the results of other groups [6,7,8]. The mobility values were just opposite to the case of the carrier concentration results. For samples A, the one grown on the 4A substrate had the highest carrier concentration, but for samples B and C without intentional nitridation, the ones grown on the 2M substrate had the highest carrier concentration. The carrier concentration for sample B grown on the 2M substrate had the best X-ray FWHM, and its surface roughness value was the highest. To understand why the B samples had such a high unintentional doping carrier concentration compared to samples A and C, we counted the hexagonal island density after hot KOH etching. The relationship between the carrier concentration and island density is shown in Figure 4e. It can clearly be seen that the unintentional doping carrier concentration decreased with the increase in island density. The polarity of the residual hexagonal islands was Ga-polar [6,9]. Thus, the island density can be thought of as proportional to the density of inversion domains in N-polar GaN film. What is already clear is that the unintentional doping carrier concentration of N-polar GaN was higher than that for Ga-polar GaN [14], because N-polar GaN film contains more O impurity than Ga-polar film, and the O_N_ defect is considered to be the main shallow donor [13,21]. As can be seen from the above analysis, the more inversion domains in N-polar GaN film, namely, the more Ga-polar domains, the less O impurities introduced in the growth process and, thus, the lower carrier concentration. The mobility of the B samples was the lowest because of the intense scattering by the high-concentration O_N_ defect. The O_N_ defect can be reduced by increasing temperature (enhance the decomposition of NH_3_) and V/III ratio during HT GaN film growth, which generates more N adatoms to compete with O adatoms [22,23]. In addition, the dislocation core can act as acceptor to compensate n-type carriers, so the B samples with the best crystal quality had the least dislocation density and, thus, the weakest compensation effect [24]. The PL results in Figure 4d indicate that the deep acceptor (V_Ga_) concentration of the B samples was the lowest, and the compensation effect for the background electron was also the lowest.

**Figure 5:**
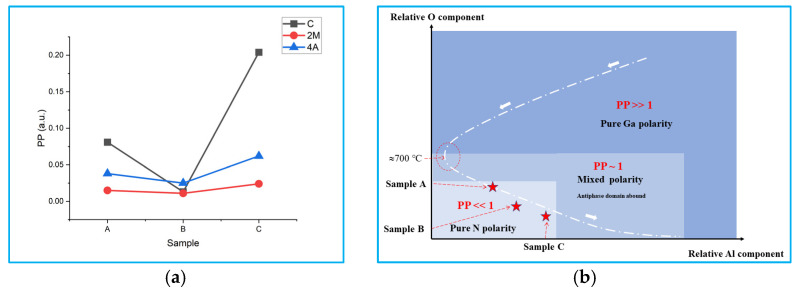
The calculated PP values of all samples: (**a**) polarity phase diagram of GaN film grown on a sapphire substrate and the relative O and Al components during the nitriding process; (**b**) the white, dotted line is the intentional nitriding curve, and the white arrows point to the direction of the increasing nitriding temperature. The positions of the three samples are marked by red five-pointed stars.

According to the above characterization results, it can be seen that at the initial stage of intentional or unintentional nitriding process (1 min), different flow rates of TMAl and NH_3_ sources had a huge impact on the quality of the subsequent N-polar GaN film. Before we consider the reason behind this, it should be emphasized the fact that a perfect N-polar GaN film containing no inversion domains is almost inexistent and some Ga-polar domains are always more or less present in the film. Thus, we defined a concept called “polarity purity” (PP) that is an area ratio of the Ga-polar to N-polar domain. When the PP value approaches 0, it corresponds to a perfect N-polar film; when the PP value is near 1, the film has a mixed polarity; when the PP value approaches infinity, it corresponds to a perfect Ga-polar film. The PP value can be expressed as:

$$\mathrm{PP}=\frac{n\times a}{S-n\times a}=\frac{\frac{n}{S}a}{1-\frac{n}{S}a}$$

where *n* and *a* are the average number and average size of the Ga-polar inversion domain in area *S*, respectively (*n*/*S* is the island density). We assumed that the average size of the Ga-polar inversion domain (“*a*”) is proportional to the average area of the hexagonal islands that remain on the surface after KOH etching: *a* = k × S_hex_ (S_hex_ is the average area of the hexagonal islands), and we approximated this parameter as k = 1. S_hex_ can be measured from SEM images with high magnification (not shown here). Then, we obtained the PP value of every sample as shown in Figure 5a. It can be seen that the PP values for all samples were less than 0.2, which indicates a dominant N polarity. The PP value of sample B was the least on every substrate; therefore, the B samples had the best polarity purity.

The PP value can be seen as a quantitative method to characterize the polarity purity of GaN film, and this PP value is mainly responsible for the nitriding process, which determines polarity. During the nitriding process, the PP value is a function of many variables such as the relative components of Al, O, and N; temperature; pressure: PP = PP (Al, O, N, T, and P). The temperature and pressure were fixed in the three experiments; thus, PP = PP (Al, O, and N). The component of N adatoms was only related to the flow rate of NH_3_ and the temperature. Thus, the relative component of N adatoms was a constant and would not change with the flow rate of TMAl because of the fixed NH_3_ flow rate and temperature in this experiment. Therefore, only the relative components of Al and O were crucial for the polarity purity of the subsequent GaN film. There are two sources for O atoms: one comes from the diffusion of oxygen atoms from inside the sapphire substrate (Al_2_O_3_) to the surface and the other comes from gas sources (such as NH_3_, N_2_, or H_2_) because of impurities (i.e., O_2_ and H_2_O) [21]. The Al atoms came from the sapphire substrate and the III-type source, TMAl. Previously, N. Stolyarchuk et al. (2018) intentionally converted the polarity of AlN from a N-polarity to a Ga-polarity by oxygen treatment. Toru et al. (2018) intentionally converted the polarity of GaN film by Al overlayer, and Man et al. (2010) converted the polarity by aluminum oxide interlayer [25,26,27]. From these reported results, we can observe that an abundance of O or Al components can convert the N polarity of GaN to a Ga polarity by forming a thin Al_9_O_3_N_7_ layer [27]. Namely, PP (Al~∞ or O~∞) >> 1, which indicates Ga polarity. Thus, a “polarity phase diagram” was created as shown in Figure 5b. According to the existing experimental reports, the key step to obtaining N-polar GaN film on a sapphire substrate is high-temperature (>700 °C) intentional nitriding, M. Sumiya and S. Fuke provided the components of the O and Al atoms that changed with the nitriding temperature in the process of intentional nitriding [28,29]. The O component gradually decreased, while the Al component first decreased and then increased with the rising nitriding temperature, and the turning point was also at approximately 700 °C. Several groups have reported that extremely high-temperature intentional nitriding can deteriorate the quality of N-polar GaN films due to the fact of inversion domains [9,27]. Based on these discussions, we created an “intentional nitriding curve” qualitatively in the polarity phase diagram, where the temperature of intentional nitriding gradually increased along the direction of where the white arrows are pointing as shown in Figure 5b. The intentional nitriding curve was based on the premise that only NH_3_ was introduced into the reaction chamber and that there was no III-type source (e.g., TMAl or TMGa), the relative values of the Al and O components changed with the change in the nitriding temperature. The closer the position came to the origin of this phase diagram, the purer the N-polarity. The probable position of samples A–C are marked as red five-pointed stars. For the A samples, which lie on the intentional nitriding curve, the O component was relatively high in this nitriding temperature. When the TMAl was put into the reaction chamber, the Al atoms and free radicals H* and CH_3_* were created by the decomposition of TMAl. The free radicals H* and CH_3_* can react with O atoms at high temperatures. Thus, the O component decreased and the Al component increased, which corresponds to the position of sample B in the polarity phase diagram (closer to the origin). Due to the TMAl source entry, the position of sample B slightly deviated from the intentional nitriding curve, and the polarity of the B sample was relatively purer. As the TMAl source continued to be added, the Al component continued to increase, and the O component continued to decrease, resulting in the position of sample C in the polarity phase diagram once again staying away from the origin, indicating that the polarity purity of the C samples decreased again. In conclusion, the B samples had the purest polarity, namely, the minimum inversion domains. Thus, the B samples had the best crystal quality, smoothest surface, and most excellent optical performance. Though the unintentional doping carrier concentration of the B samples was relatively high, it can be reduced by increasing the temperature and the V/III ratio during the HT GaN film’s growth [22,23].

## 4. Conclusions

Right before the HT AlN layer’s growth, treating sapphire substrate simultaneously with NH_3_ and a certain amount of TMAl sources obtained a higher crystal quality and surface flatness for N-polar GaN films compared with only inletting a NH_3_ source (i.e., intentional nitridation). We attributed this difference to the control of polarity by different relative components of Al and O on the sapphire substrate during the high-temperature nitriding process. A polarity phase diagram was created to illustrate this situation. The polarity “purity”, namely, the ratio of N polarity to Ga polarity of sample B was the highest, which made it the most close to a perfect N-polar GaN film. Thus, sample B had the highest crystal quality, surface roughness, and luminescence intensity. Although sample B had a higher electron concentration than samples A and C, this value was in the same order of magnitude as that of ordinary unintentional doping N-polar GaN film (10^18^ cm^−3^), which also indicates that sample B had the best polarity purity. For different vicinal sapphire substrates, N-polar GaN films grown on 2M substrate had smaller (002) and (102) X-ray rocking curve FWHM values but a larger surface RMS roughness than films grown on 4A substrate.

## Figures and Tables

**Table 1:** The source flow rate of NH_3_ and TMAl at the initial 1 min before the HT AlN layer growth.

Sample	NH_3_/sccm	TMAl/sccm
A	12,000	0
B	12,000	136
C	12,000	204

**Table 2:** Summary of the measured results for the total samples.

Sample	Substrate	(002)/(102) (Arcsec)	Carrier Concentration (10^17^ cm^−3^)	Hall Mobility (cm^2^/Vs)
**A**	C	330/1397	8.17	239
	2M	283/495	7.08	313
	4A	383/460	8.72	279
**B**	C	303/416	24.2	166
	2M	237/337	33.8	174
	4A	405/439	24.5	239
**C**	C	363/1014	5.02	252
	2M	379/500	5.95	325
	4A	378/474	3.47	361

## Data Availability

The data that support the findings of this study are available from the corresponding author upon reasonable request.
